# The role of carboxyl­ate ligand orbitals in the breathing dynamics of a metal-organic framework by resonant X-ray emission spectroscopy

**DOI:** 10.1107/S1600577524000584

**Published:** 2024-02-16

**Authors:** Ralph Ugalino, Kosuke Yamazoe, Jun Miyawaki, Hisao Kiuchi, Naoya Kurahashi, Yuka Kosegawa, Yoshihisa Harada

**Affiliations:** a Department of Advanced Materials Science, Graduate School of Frontier Sciences, The University of Tokyo, Kashiwa, Chiba 277-8561, Japan; b Institute for Solid State Physics (ISSP), The University of Tokyo, Kashiwa, Chiba 277-8561, Japan; c Institute for Advanced Synchrotron Light Source, National Institutes for Quantum and Radiological Science and Technology (QST), Sendai, Miyagi 980-8579, Japan; d Synchrotron Radiation Collaborative Research Organization, The University of Tokyo, Sendai, Miyagi 980-8572, Japan; Photon Science Innovation Center, Japan

**Keywords:** resonant X-ray emission spectroscopy, phase transition, metal-organic framework

## Abstract

Metal-organic frameworks (MOFs) exhibit structural flexibility induced by temperature and guest adsorption, as demonstrated in the structural breathing transition in certain MOFs between narrow-pore and large-pore phases. Soft modes were suggested to entropically drive such pore breathing through enhanced vibrational dynamics at high temperatures. In this work, oxygen *K*-edge resonant X-ray emission spectroscopy of the MIL-53(Al) MOF was performed to selectively probe the electronic perturbation accompanying pore breathing dynamics at the ligand carboxyl­ate site for metal–ligand interaction. It was observed that the temperature-induced vibrational dynamics involves switching occupancy between antisymmetric and symmetric configurations of the carboxyl­ate oxygen lone pair orbitals, through which electron density around carboxyl­ate oxygen sites is redistributed and metal–ligand interactions are tuned. In turn, water adsorption involves an additional perturbation of π orbitals not observed in the structural change solely induced by temperature.

## Abstract 2

Orbital occupancy of the ligand carboxyl­ate in-plane lone pair orbitals modulates the vibrational dynamics of certain breathing modes responsible for the structural phase transition of a carboxyl­ate metal-organic framework.

## 1. Introduction

**Figure 1:**
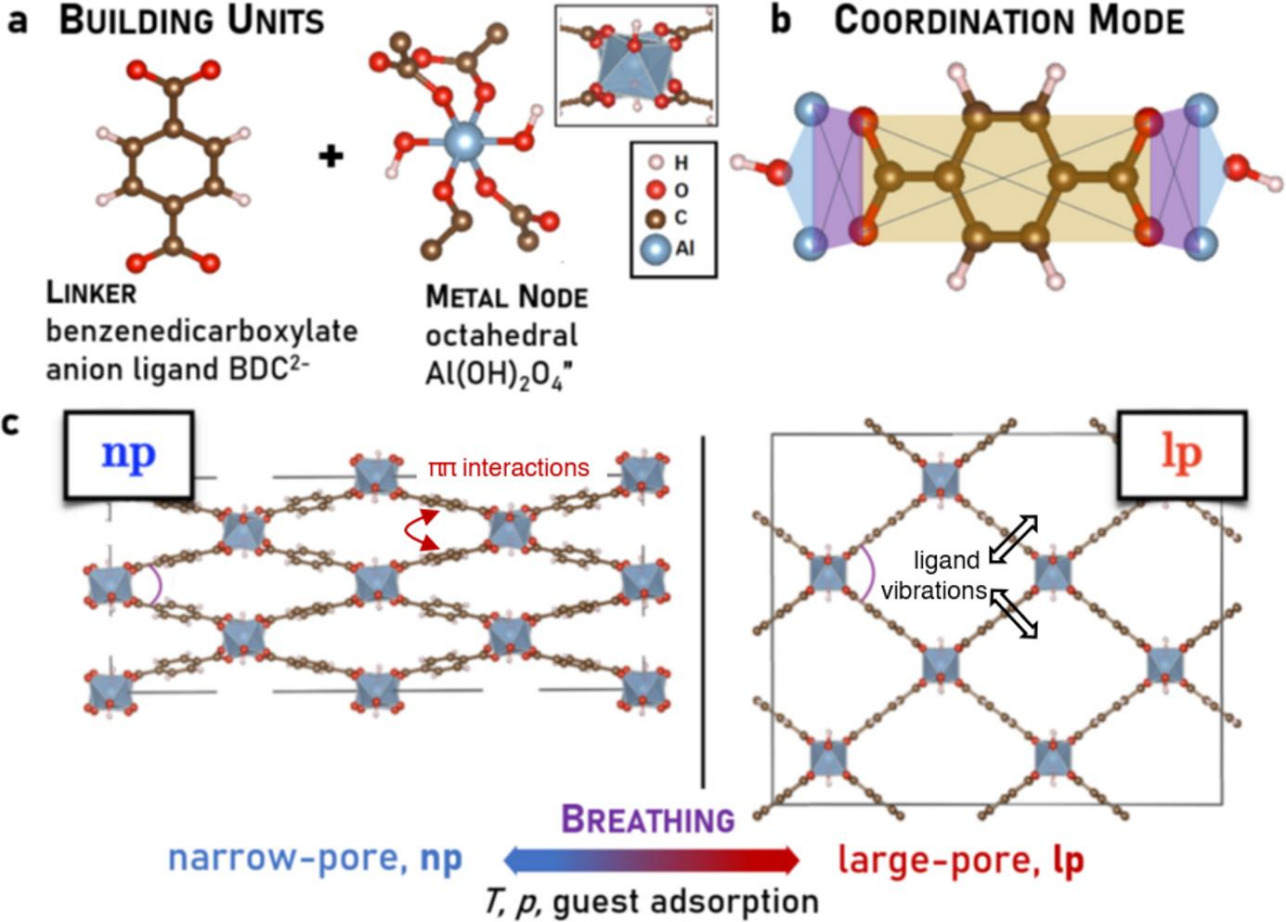
Structural building units (*a*) of the target MIL-53(Al) metal-organic framework (MOF): the benzene­dicarboxyl­ate (BDC) linker and the octahedral aluminium-oxo metal node (inset). Coordination geometry (*b*) where the ligand carboxyl­ate group (COO) bridges adjacent metal centers. Crystal structures (*c*) of the narrow-pore, np, and the large-pore, lp, forms of a MIL-53 MOF.

Metal-organic frameworks (MOFs) are soft porous crystals with dynamic crystalline frameworks (Horike *et al.*, 2009 ▸; Coudert *et al.*, 2013 ▸) exhibiting reversible structural transformations in response to external stimuli. A noteworthy example is the breathing transition observed in the MIL-53 family of MOFs, a structural phase transition (Loiseau *et al.*, 2004 ▸; Liu *et al.*, 2008 ▸) between large-pore and narrow-pore forms which can be induced by temperature and guest adsorption. The MIL-53 structure [Fig. 1 ▸(*a*)] consists of octahedral aluminium oxo metal nodes, bridged through terephthalate or benzene­dicarboxyl­ate (BDC) ligands, and assembled into a characteristic wine-rack framework topology (Loiseau *et al.*, 2004 ▸; Liu *et al.*, 2008 ▸). The carboxyl­ate functionality, COO, is a defining feature of MOF structures, and its ability to bridge metal sites [Fig. 1 ▸(*b*)] enables a variety of framework topologies [Fig. 1 ▸(*c*)] and holds the MOF structure in place.

Free energy calculations (Walker *et al.*, 2010 ▸; Wieme *et al.*, 2018 ▸) suggested that the thermodynamic stability of narrow-pore and large-pore phases of a MIL-53 MOF depends on the interplay between long-range dispersion interactions and vibrational entropy. The narrow-pore phase [Fig. 1 ▸(*c*)], observed at low temperature, is stabilized by ππ stacking interactions between the ligand aromatic moieties that tend to favor pore collapse (Walker *et al.*, 2010 ▸; Wieme *et al.*, 2018 ▸; Grinnell & Samokhvalov, 2018 ▸). In turn, the large-pore phase [Fig. 1 ▸(*c*)], observed at high temperature, is stabilized by vibrational entropy arising from enhanced ligand vibrational dynamics afforded with the larger pore volume (Walker *et al.*, 2010 ▸; Wieme *et al.*, 2018 ▸). Previous work (Liu *et al.*, 2008 ▸; Salazar *et al.*, 2015 ▸; Hoffman *et al.*, 2018 ▸) showed that pore collapse at low temperatures was accompanied by a decrease in vibrational energy, or softening, of certain vibrational modes such as carboxyl­ate asymmetric stretching (Salazar *et al.*, 2015 ▸; Hoffman *et al.*, 2018 ▸), benzene ring libration and linker twisting (Liu *et al.*, 2008 ▸) modes. Enhancing the vibrational dynamics of these soft modes was proposed to drive pore breathing into large-pore phases at higher temperatures (Liu *et al.*, 2008 ▸; Walker *et al.*, 2010 ▸; Bersuker, 2013 ▸; Salazar *et al.*, 2015 ▸; Wieme *et al.*, 2018 ▸; Hoffman *et al.*, 2018 ▸; Bersuker, 2021 ▸). The apparent universality of such mode softening, especially for the carboxyl­ate stretching modes, was observed across carboxyl­ate-based MOFs (Andreeva *et al.*, 2020 ▸) as an indirect measure of the strength of MOF metal–ligand interaction. Moreover, it was also suggested how guest adsorption could stabilize the narrow-pore over the large-pore phase via the adsorption interaction (Coudert *et al.*, 2008 ▸, 2014 ▸). However, there are aspects of structural transitions in MOFs that remain unresolved solely on thermodynamic grounds. These include the onset of ligand defect site formation in UiO-66 MOFs (Shearer *et al.*, 2014 ▸), and of interpenetration in MOFs with very large ligands (Bara *et al.*, 2019 ▸), which become favored, instead of pore collapse, at low temperatures. Within just the MIL-53 family of MOFs, despite sharing an identical framework topology, changing the metal center, say from Al to Fe or Ga (Volkringer *et al.*, 2009 ▸), shifts the breathing transition temperature by large jumps which cannot be accounted for solely by ion size effects, and the role of metal–ligand orbital interaction appears to be significant. Understanding the interplay of temperature and guest adsorption for structural changes in MOFs, including the contribution of metal–ligand orbital interaction, is key to designing nanoporous materials with stimuli-responsive phase transitions exhibiting practical reversibility for real-time applications.

Resonant X-ray emission spectroscopy (RXES) is an emerging method for probing valence electronic states of small molecules (Tokushima *et al.*, 2009 ▸; Horikawa *et al.*, 2009 ▸; Meyer *et al.*, 2014 ▸; Eckert *et al.*, 2022 ▸) with element and symmetry selectivity. While nonresonant X-ray emission spectroscopy (XES) probes the entire manifold of occupied orbitals, resonant excitation under RXES imposes symmetry restrictions such that only a few selected occupied orbitals are observed in the spectra. In particular, the symmetry of the unoccupied orbital accessed during resonant excitation determines whether certain emission channels will be allowed or forbidden (Monson & McClain, 1970 ▸; Gel’mukhanov & Ågren, 1994 ▸; Meyer *et al.*, 2014 ▸; Miyawaki *et al.*, 2017 ▸; Eckert *et al.*, 2022 ▸). In this work, oxygen *K*-edge RXES was undertaken for the MOF, MIL-53(Al), in order to selectively probe the carboxyl­ate ligand orbitals participating in the metal–ligand interaction, both in the presence and absence of adsorbed water, and elucidate their role in modulating MOF vibrational dynamics responsible for pore breathing. RXES measurements were performed using the high-resolution soft X-ray emission spectrometer at the SPring-8 BL07LSU HORNET endstation (Harada *et al.*, 2012 ▸; Yamamoto *et al.*, 2014 ▸). Electronic structure calculations on the benzene­dicarboxyl­ate anion ligand were undertaken to adequately account for the spectral features which exhibited change with temperature and water adsorption.

## 2. Results and discussion

### 2.1. X-ray absorption spectroscopy (XAS)

**Figure 2:**
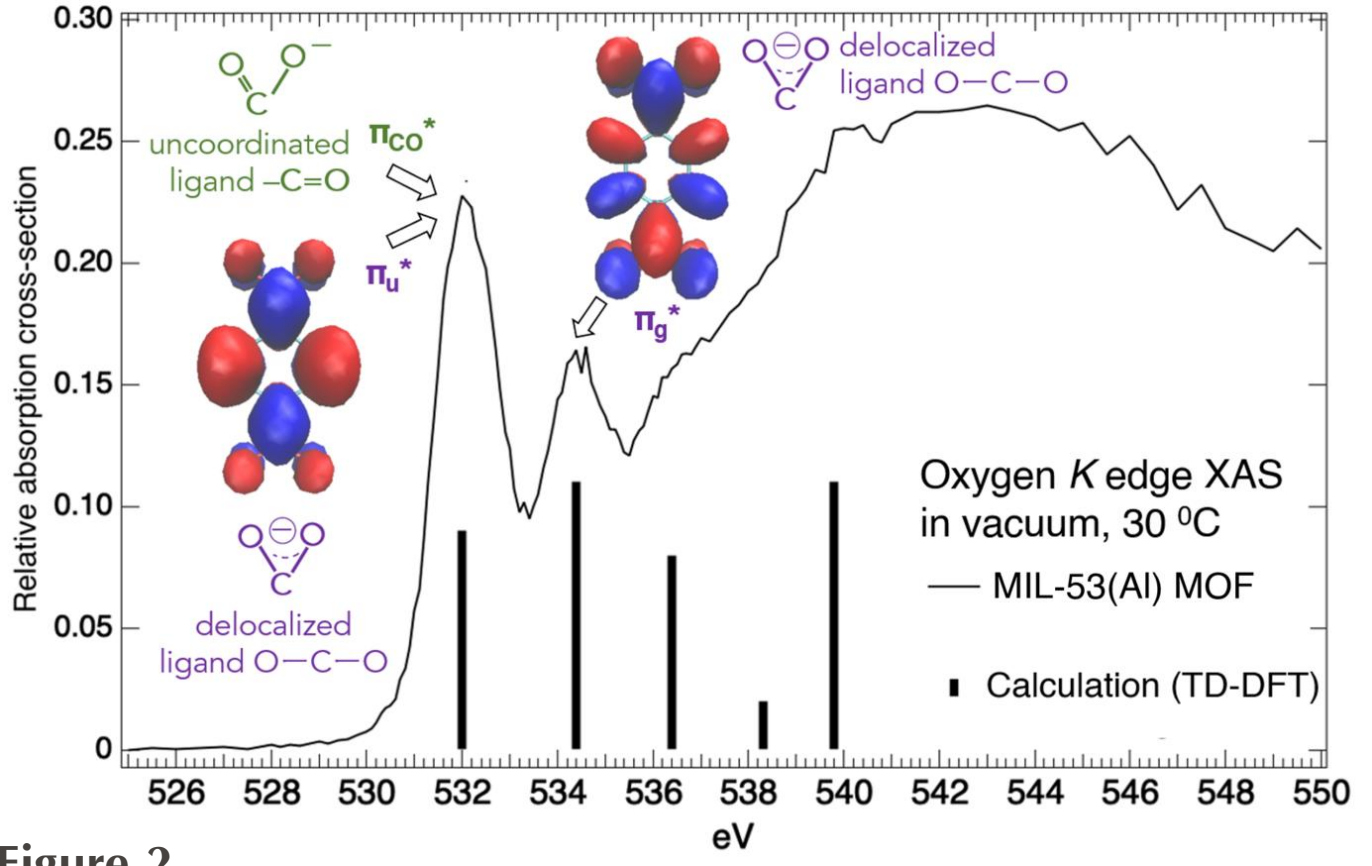
Oxygen *K*-edge XAS for MIL-53(Al) MOF, in vacuum at 30°C, compared with TD-DFT calculated XAS energies for the benzene­dicarboxyl­ate anion ligand, with the unoccupied orbitals 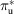 , 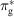 and 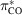 assigned to the pre-edge features.

Oxygen *K*-edge XAS of MIL-53(Al) MOF, in vacuum at 30°C, showed two pre-edge features (Fig. 2 ▸) at 532.0 and 534.4 eV. Time-dependent density functional theory (TD-DFT) XAS calculations on the benzendi­carboxyl­ate (BDC) anion ligand suggested that the 532.0 and 534.4 eV pre-edge peaks arise from the unoccupied orbitals 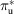 and 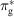 , respectively. While both are derived from the same delocalized carboxyl­ate COO antibonding 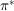 fragment orbital, they involve different benzene group orbitals which created the ∼2.4 eV energy gap (Hennies *et al.*, 2007 ▸) between the 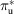 and 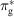 states. Moreover, it is noted that the 532.0 eV pre-edge peak includes contribution from the antibonding 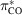 orbital for the localized carbonyl C=O group, as was observed in carboxyl­ates and amino acids (Tokushima *et al.*, 2009 ▸; Horikawa *et al.*, 2009 ▸; Meyer *et al.*, 2014 ▸; Eckert *et al.*, 2022 ▸). Hence, RXES measurements were opted at 534.4 eV excitation, instead of at 532.0 eV, in order to probe the delocalized carboxyl­ate COO units (Fig. 2 ▸) involved in bridging metal sites within the MOF structure. This minimizes the contribution of localized carbonyl C=O groups indicative of uncoordinated ligand sites in the subsequent RXES spectra.

### 2.2. Resonant X-ray emission spectroscopy (RXES)

**Figure 3:**
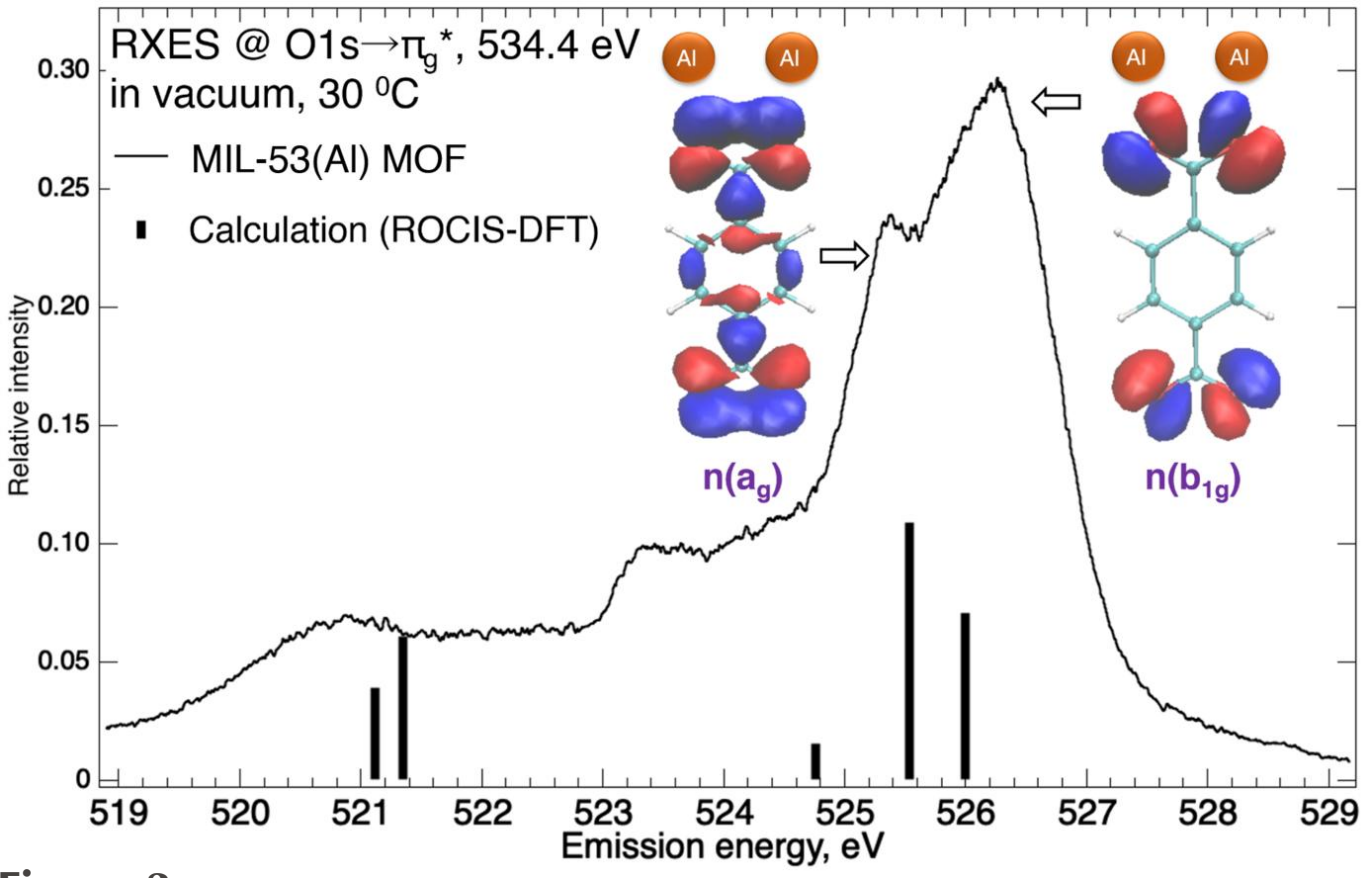
RXES of MIL-53(Al) MOF at 534.4 eV excitation (O1*s* → 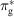 ), in vacuum at 30°C, compared with ROCIS-DFT calculated emission energies for the benzene­dicarboxyl­ate anion ligand, along with the occupied orbitals, *n*(*b*_1g_) and *n*(*a*_g_), derived from the ligand carboxyl­ate oxygen lone pair orbitals in either antisymmetric *b*_1g_ or symmetric *a*_g_ configuration.

RXES (Fig. 3 ▸) at the O 1*s* → 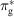 excitation at 534.4 eV (Fig. 2 ▸) for the MIL-53(Al) MOF was measured in vacuum at 30°C (Fig. 3 ▸). RXES calculations (Roemelt *et al.*, 2013 ▸), under restricted open configuration interaction with single excitations using DFT-derived orbitals (ROCIS-DFT) for the BDC anion ligand, suggested that the highest-lying emission features at 526.2 and 525.4 eV arise from the *n*(*b*_1g_) and *n*(*a*_g_) states (Fig. 3 ▸), respectively, derived from the carboxyl­ate COO oxygen in-plane lone pair orbitals. The calculated ∼0.4 eV energy gap between these two states for the free BDC anion ligand was attributed to the difference in orbital overlap between the in-plane lone pair orbitals of the two oxygen atoms on the COO carboxyl­ate group, in either an antisymmetric *b*_1g_ or a symmetric *a*_g_ configuration (Eckert *et al.*, 2022 ▸). In *n*(*b*_1g_), the antibonding-like interaction between the lone pair orbitals creates a region of reduced electron density between the carboxyl­ate oxygen sites, along with a diffuse region of electron density distributed away from the oxygen sites and directed separately into the flanking metal centers (Fig. 3 ▸). In turn, in *n*(*a*_g_), the bonding-like interaction between the lone pair orbitals concentrates electron density within the region between the carboxyl­ate oxygens, favoring shared interaction with the neighboring metal centers (Fig. 3 ▸). The deep-lying weak emission feature at ∼521.4 eV was assigned to out-of-plane carboxyl­ate π orbitals which delocalize into the neighboring benzene ring π system. The emission bands unaccounted for in the ligand RXES calculations are attributed to contributions from the oxide oxygens in the aluminium oxo centers (Ertan *et al.*, 2017 ▸).

### 2.3. The role of temperature

**Figure 4:**
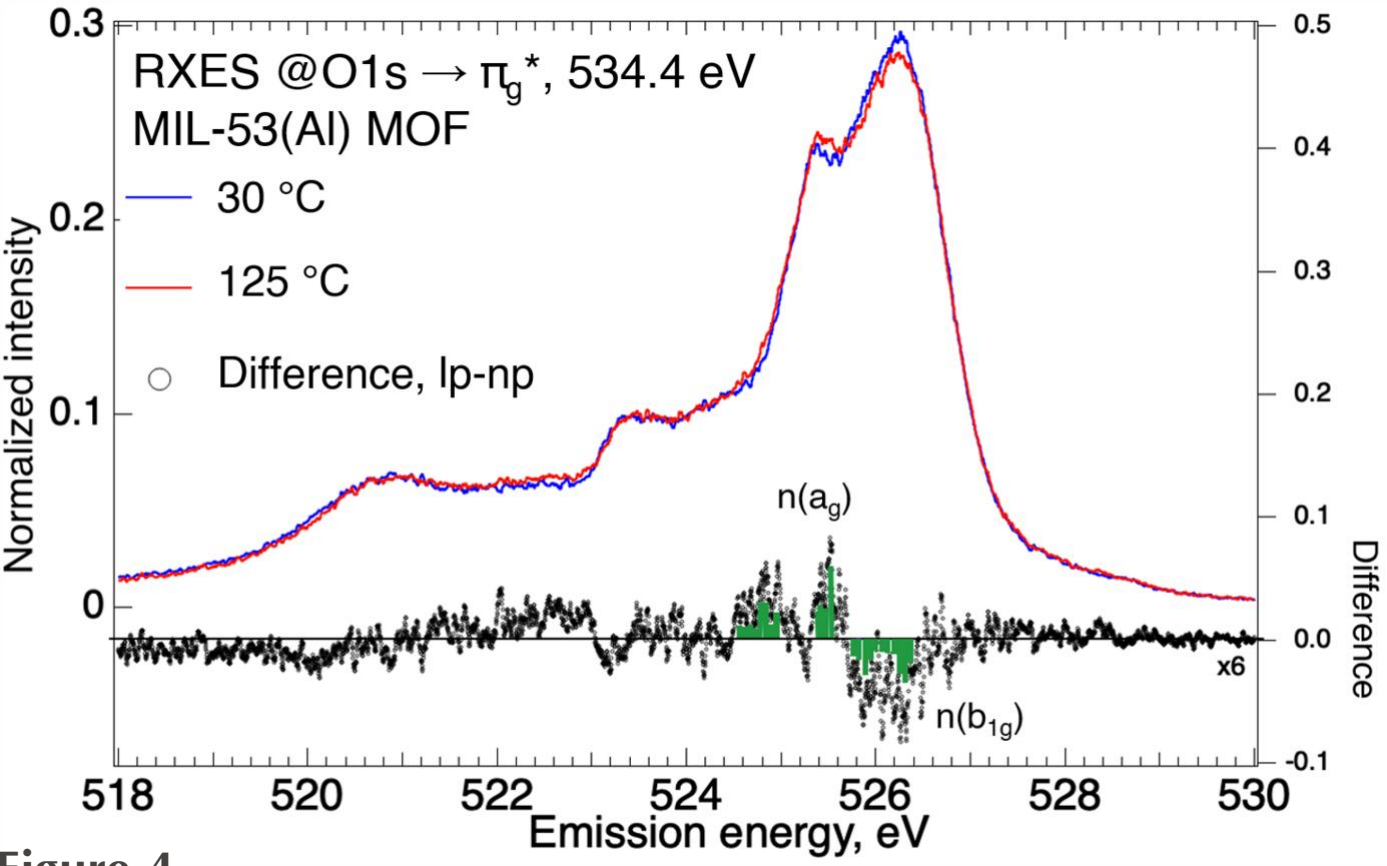
RXES at 534.4 eV excitation (O1*s* → 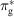 ) for the MIL-53(Al) MOF in vacuum, at 30°C and 125°C.

The temperature dependence of the RXES spectra at 534.4 eV excitation (Fig. 4 ▸) for the MIL-53(Al) MOF showed modulation of emission intensities for the highest-lying valence states, with a reduced emission at 526.2 eV [*n*(*b*_1g_)] compensated by an enhanced emission at 525.4 eV [*n*(*a*_g_)] upon temperature increase. Such electronic perturbation appears to be involved in the structural change accompanying the onset of pore breathing at higher temperatures (Loiseau *et al.*, 2004 ▸; Liu *et al.*, 2008 ▸; Volkringer *et al.*, 2009 ▸). This includes the slightly shorter carboxyl­ate C—O bond inferred from the blue shift [Fig. S1 of the supporting information (SI)] for the stretching mode, and the modest increase in lattice constant (SI, Fig. S2) especially across the pore walls. The RXES spectra suggest that pore breathing, solely induced by temperature increase under vacuum, is accompanied by a modulation of orbital occupancy of *n*(*b*_1g_) and *n*(*a*_g_) states, as reflected in the change in their relative emission intensities.

**Figure 5:**
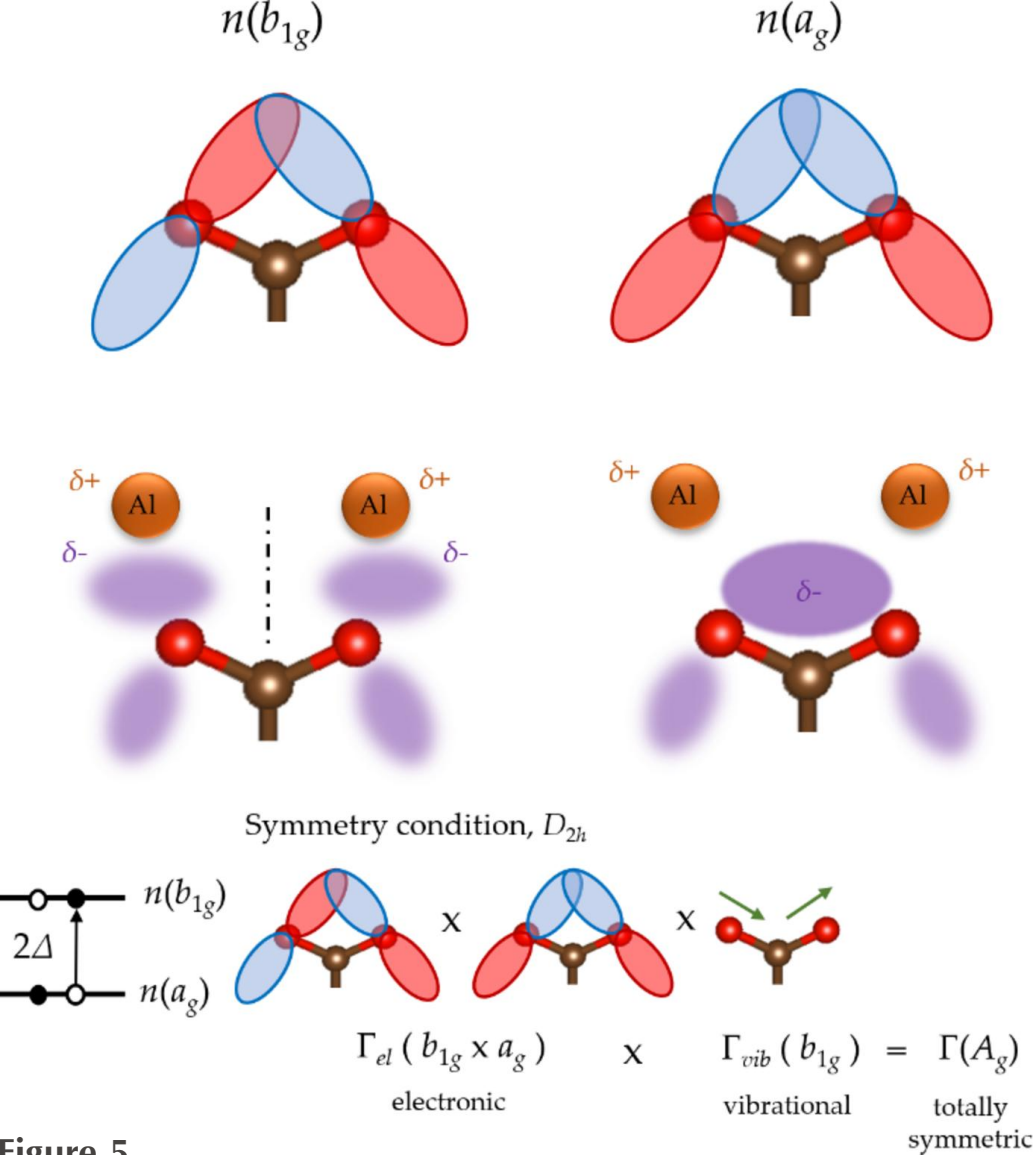
Pseudo-Jahn-Teller mechanism for mode softening in a carboxyl­ate MOF by tuning the occupancy of *n*(*b*_1g_) and *n*(*a*_g_) lone pair orbitals.

Ligand vibrational dynamics in carboxyl­ate MOFs is closely related to the strength of the metal–ligand (*M*−O) interaction (Andreeva *et al.*, 2020 ▸), with ‘loose’ *M*—O bond populations preferred over ‘tight’ ones, and stabilized by entropy at higher temperatures (Walker *et al.*, 2010 ▸: Wieme *et al.*, 2018 ▸). Tuning the orbital population for the *n*(*b*_1g_) and *n*(*a*_g_) states (Fig. 5 ▸) is one mechanism towards modulating the strength of metal–ligand interaction, and the accompanying ligand vibrational dynamics and entropic stabilization. In this mechanism, *n*(*b*_1g_) orbital occupation at low temperature appears to be a precedent for carboxyl­ate C—O bond inequivalence, as observed in the narrow-pore phase, as regions of electron density are directed towards the flanking metal centers separately due to the nodal plane between the carboxyl­ate oxygens. In turn, *n*(*a*_g_) orbital occupation at high temperature appears to be a precedent for carboxyl­ate C—O bond equivalence, as observed in the large-pore phase, owing to the shared interaction of the overlapping electron density regions with the neighboring metal centers.

A pseudo-Jahn-Teller (PJT) description of the modulation of orbital occupancy (Bersuker, 2013 ▸, 2021 ▸) is applied for the *n*(*b*_1g_) and *n*(*a*_g_) orbital populations of the MOF carboxyl­ate upon temperature change. In the PJT mechanism, orbital populations can change via orbital mixing mediated by coupling these electronic states, Γ_el_(*b*_1g_) and Γ_el_(*a*_g_), to a vibrational mode, Γ_vib_(*b*_1g_), under the *D*_2*h*_ point group symmetry of the BDC anion ligand, that satisfies the symmetry condition Γ_el_(*b*_1g_) × Γ_el_(*a*_g_) × Γ_vib_(*b*_1g_) = Γ(*A*_g_). In particular, the carboxyl­ate asymmetric stretching mode of the BDC ligand of *b*_1g_ symmetry (Fig. 5 ▸) is taken to participate in this mechanism, as it is sensitive (SI, Fig. S1) to structural changes in MOFs (Salazar *et al.*, 2015 ▸; Hoffman *et al.*, 2018 ▸). Also, being an in-plane vibrational mode, the carboxyl­ate *b*_1g_ asymmetric stretching mode has a large spatial overlap with the in-plane *n*(*b*_1g_) and *n*(*a*_g_) lone pair orbitals being mixed, enhancing the PJT effect as a result (Sato *et al.*, 2006 ▸; Bersuker, 2013 ▸, 2021 ▸). Finally, it is remarked that while the microscopic mechanism of pore breathing in MIL-53 MOFs has been tackled on entropic and mechanical grounds (Walker *et al.*, 2010 ▸; Triguero *et al.*, 2011 ▸; Cockayne, 2017 ▸; Wieme *et al.*, 2018 ▸), the modulation of orbital occupancy (Fig. 5 ▸) elaborated in this work involves an earlier stage and a smaller scale of the structural phase transition, just at the onset of ‘loosening’ or ‘tightening’ the metal–ligand interaction that precedes the collective ligand motion needed for the drastic change in lattice structure during the breathing transition.

### 2.4. The role of water adsorption

**Figure 6:**
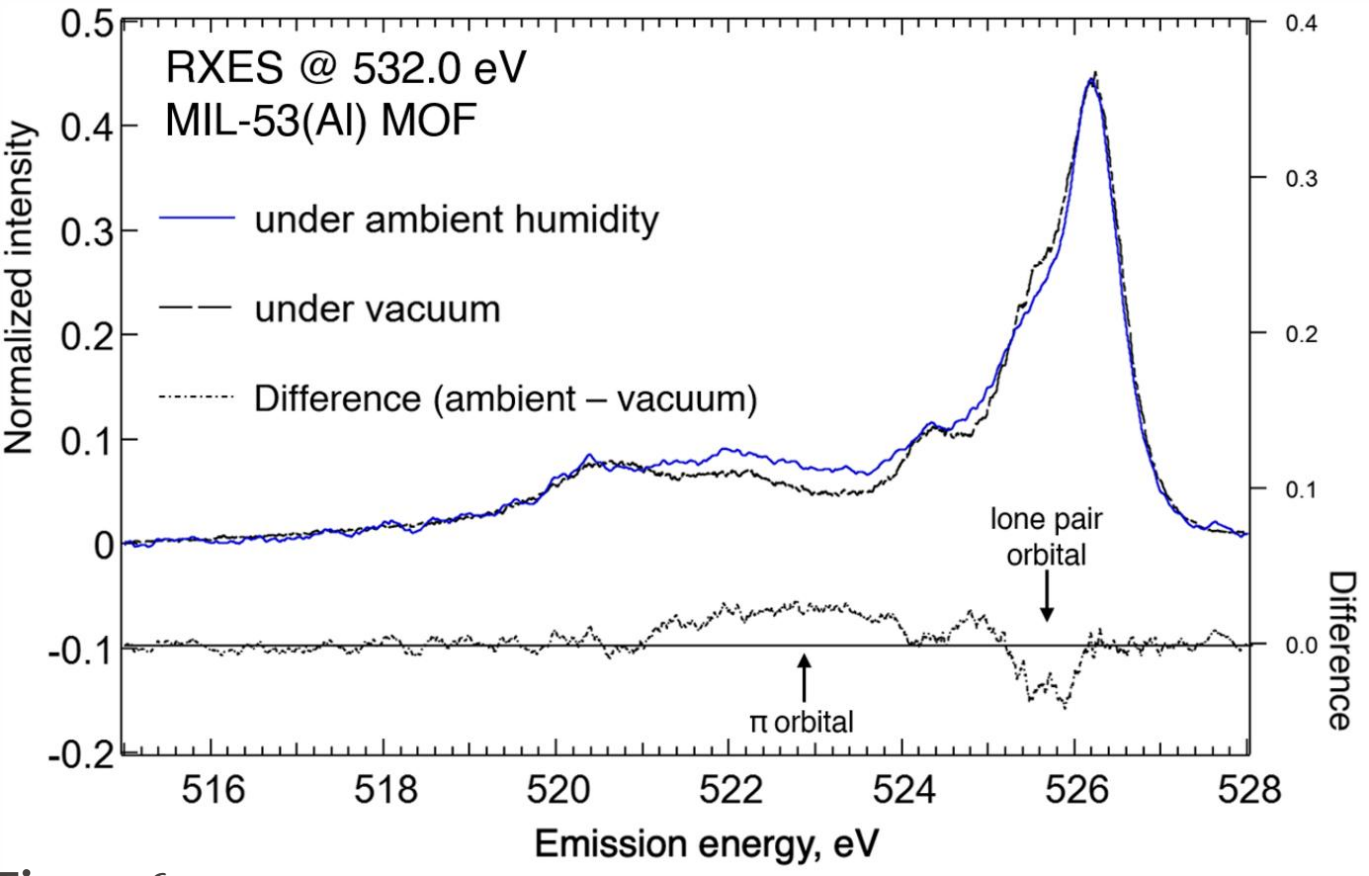
RXES at 532.0 eV excitation for the MIL-53(Al) MOF at 30°C under 60% relative humidity.

The effect of water adsorption on the RXES spectra (Fig. 6 ▸) at 532.0 eV excitation (SI, Fig. S3) at 30°C showed that, upon MOF hydration, reduced emission at 525.4 eV is compensated by enhanced emission at 522.0 eV. These emission features at 525.4 and 522.0 eV derive from the carboxyl­ate oxygen in-plane lone pair and out-of-plane π orbitals, respectively (Horikawa *et al.*, 2009 ▸; Meyer *et al.*, 2014 ▸; Eckert *et al.*, 2022 ▸). Such orbital modulation suggests how water adsorption can perturb the out-of-plane electron density by accessing deep-lying π orbitals, which was not observed (SI, Fig. S4) for pore breathing in vacuum solely induced by temperature. While a similar RXES behavior at 534.4 eV excitation was anticipated, this excitation energy already overlaps with the absorption pre-edge for the water molecule (Horikawa *et al.*, 2009 ▸; Meyer *et al.*, 2014 ▸; Eckert *et al.*, 2022 ▸). Subtracting the contribution of adsorbed water would be difficult such that, ultimately, RXES at 532.0 eV excitation was opted for in this case. It is remarked how such a difference in orbital occupancy observed under vacuum and ambient conditions could provide alternative pathways for pore breathing, as exemplified in their distinct breathing kinetics, with a facile pore collapse under ambient conditions (Loiseau *et al.*, 2004 ▸) compared with a severe hysteresis behavior under vacuum (Liu *et al.*, 2008 ▸).

## 3. Conclusion

In summary, electronic perturbation at the ligand carboxyl­ate accompanying pore breathing in the metal-organic framework MIL-53(Al) was observed by oxygen *K*-edge resonant X-ray emission spectroscopy. Pore breathing in vacuum, solely induced by temperature, involved modulation of orbital occupancy of carboxyl­ate oxygen in-plane lone pair orbitals in either an antisymmetric or a symmetric configuration. In turn, water adsorption into the MOF involved additional perturbation of out-of-plane π orbitals. More than a mere counterion, the carboxyl­ate ligand bears an electronic structure motif that is intrinsically functional for driving structural change in MOFs. Tailoring the symmetry of the ligand carboxyl­ate electronic states appears to be a potential route towards the design of novel functional MOFs with controllable structural transitions.

## 4. Related literature

The following references, not cited in the main body of the paper, have been cited in the supporting information: Becke (1993 ▸); Chmela & Harding (2018 ▸); Drisdell *et al.* (2013 ▸); Kang *et al.* (2011 ▸); Momma & Izumi (2008 ▸); Neese (2012 ▸); Stephens *et al.* (1994 ▸); Weigend & Ahlrichs (2005 ▸).

## Supplementary Material

Supporting information file. DOI: 10.1107/S1600577524000584/iy5002sup77.docx
