# Supplementary material for: The role of carboxyl­ate ligand orbitals in the breathing dynamics of a metal-organic framework by resonant X-ray emission spectroscopy
Source: J Synchrotron Radiat. 2024 Feb 16;31(Pt 2):217–21. doi: 10.1107/S1600577524000584 (PMC10914173; doi:10.1107/S1600577524000584)
Supplement: Supplementary file 1 [file s-31-00217-sup77.docx]

The role of carboxylate ligand orbitals in the breathing dynamics of a metal-organic framework by resonant x-ray emission spectroscopy (RXES)

**Ralph Ugalino^ab^*, Kosuke Yamazoe^b^, Jun Miyawaki^c^, Hisao Kiuchi^ab^, Naoya Kurahashi^b^, Yuka Kosegawa^b^ and Yoshihisa Harada^abd^***

^a^Department of Advanced Materials Science, Graduate School of Frontier Sciences, The University of Tokyo, Kashiwa, Chiba, 277-8561, Japan
^b^Institute for Solid State Physics (ISSP), The University of Tokyo, Kashiwa, Chiba, 277-8561, Japan
^c^Institute for Advanced Synchrotron Light Source, National Institutes for Quantum and Radiological Science and Technology (QST), Sendai, Miyagi, 980-8579, Japan
^d^Synchrotron Radiation Collaborative Research Organization, The University of Tokyo, Sendai, Miyagi, 980-8572, Japan

Correspondence email: ugalino@issp.u-tokyo.ac.jp; harada@issp.u-tokyo.ac.jp

1. Orbital occupancy of the ligand carboxylate in-plane lone pair orbitals modulates the vibrational dynamics of certain breathing modes responsible for the structural phase transition of a carboxylate metal-organic framework.
2. Metal-organic frameworks (MOFs) exhibit structural flexibility induced by temperature and guest adsorption, as demonstrated in the structural breathing transition in certain MOFs between narrow-pore and large-pore phases. Soft modes were suggested to entropically drive such pore breathing through enhanced vibrational dynamics at high temperatures. In this work, oxygen *K* edge resonant x-ray emission spectroscopy (RXES) for the MIL-53(Al) MOF was performed to selectively probe the electronic perturbation accompanying pore breathing dynamics at the ligand carboxylate site for metal-ligand interaction. It was observed that the temperature-induced vibrational dynamics involves switching occupancy between antisymmetric and symmetric configurations of the carboxylate oxygen lone pair orbitals, through which electron density around carboxylate oxygen sites is redistributed and metal-ligand interaction is tuned. In turn, water adsorption involves an additional perturbation of π orbitals not observed in the structural change solely induced by temperature.
3. resonant x-ray emission spectroscopy; phase transition; metal-organic framework
4. Introduction

Metal-organic frameworks (MOFs) are soft porous crystals with dynamic crystalline frameworks (Horike *et al.,* 2009 ; Coudert *et al.*, 2013) exhibiting reversible structural transformations in response to external stimuli. A noteworthy example is the breathing transition observed in the MIL-53 family of MOFs, a structural phase transition (Loiseau *et al.*, 2004 ; Liu *et al.*, 2008) between large-pore and narrow-pore forms which can be induced by temperature and guest adsorption. The MIL-53 structure (Fig. 1a) consists of octahedral aluminum oxo metal nodes, bridged through terephthalate or benzenedicarboxylate (BDC) ligands, and assembled into a characteristic wine-rack framework topology (Loiseau *et al.*, 2004 ; Liu *et al.*, 2008). The carboxylate functionality, COO, is a defining feature of MOF structures, and its ability to bridge metal sites (Fig. 1b) enables a variety of framework topologies (Fig. 1c) and holds the MOF structure in place.


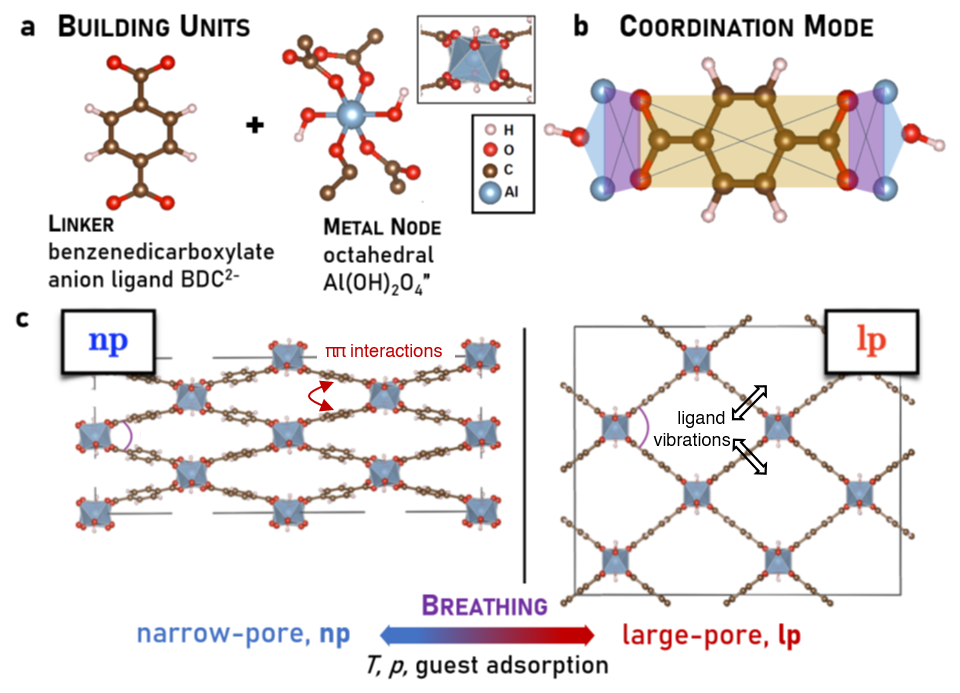


1. Structural building units (*a*) of the target MIL-53(Al) metal-organic framework (MOF): the benzenedicarboxylate (BDC) linker and the octahedral aluminum-oxo metal node (*inset*). Coordination geometry (*b*) where the ligand carboxylate group (COO) bridges adjacent metal centers. Crystal structures (*c*) of the narrow-pore, **np**, and the large-pore, **lp**, forms of a MIL-53 MOF.

Free energy calculations (Walker *et al*., 2010 ; Wieme *et al.*, 2018) suggested that the thermodynamic stability of narrow-pore and large-pore phases of a MIL-53 MOF depends on the interplay between long-range dispersion interactions and vibrational entropy. The narrow-pore phase (Fig. 1c), observed at low temperature, is stabilized by ππ stacking interactions between the ligand aromatic moieties that tend to favor pore collapse (Walker *et al*., 2010 ; Wieme *et al.*, 2018 ; Grinnell *et al*., 2018). In turn, the large-pore phase (Fig. 1c), observed at high temperature, is stabilized by vibrational entropy arising from enhanced ligand vibrational dynamics afforded with the larger pore volume (Walker *et al*., 2010 ; Wieme *et al.*, 2018). Previous work (Liu *et al.*, 2008 ; Salazar *et al*., 2015 ; Hoffmann *et al.*, 2018) showed that pore collapse at low temperatures was accompanied by a decrease in vibrational energy, or softening, of certain vibrational modes such as carboxylate asymmetric stretching (Salazar *et al*., 2015 ; Hoffmann *et al.*, 2018 ), benzene ring libration, and linker twisting (Liu *et al.*, 2008) modes. Enhancing the vibrational dynamics of these soft modes was proposed to drive pore breathing into large-pore phases at higher temperatures (Liu *et al.*, 2008 ; Walker *et al*., 2010 ; Bersuker, 2013 ; Salazar *et al.*, 2015 ; Wieme *et al.*, 2018 ; Hoffmann *et al.*, 2018 ; Bersuker, 2021). The apparent universality of such mode softening, especially for the carboxylate stretching modes, was observed across carboxylate-　based MOFs (Andreeva *et al*., 2020) as an indirect measure of the strength of MOF metal-ligand interaction. Moreover, it was also suggested how guest adsorption could stabilize the narrow-pore over the large-pore phase via the adsorption interaction (Coudert *et al*., 2008 ; Coudert *et al*., 2014). However, there are aspects of structural transitions in MOFs that remain unresolved solely on thermodynamic grounds. These include the onset of ligand defect site formation in UiO-66 MOFs (Shearer *et al*., 2014), and of interpenetration in MOFs with very large ligands (Bara *et al*., 2019), which become favored, instead of pore collapse, at low temperatures. Within just the MIL-53 family of MOFs, despite sharing an identical framework topology, changing the metal center, say from Al to Fe or Ga (Volkringer *et al*., 2009), shifts the breathing transition temperature by large jumps which cannot be accounted for solely by ion size effects, and the role of metal-ligand orbital interaction appears to be significant. Understanding the interplay of temperature and guest adsorption for structural changes in MOFs, including the contribution of metal-ligand orbital interaction, is key to designing nanoporous materials with stimuli-responsive phase transitions exhibiting practical reversibility for real-time applications.

Resonant x-ray emission spectroscopy (RXES) is an emerging method for probing valence electronic states of small molecules (Tokushima *et al*., 2009 ; Horikawa *et al*., 2009 ; Meyer *et al*., 2014 ; Eckert *et al*., 2022) with element and symmetry selectivity. While nonresonant x-ray emission spectroscopy (XES) probes the entire manifold of occupied orbitals, resonant excitation under RXES imposes symmetry restrictions such that only a few selected occupied orbitals are observed in the spectra. In particular, the symmetry of the unoccupied orbital accessed during resonant excitation determines whether certain emission channels will be allowed or forbidden (Monson *et al.*, 1970 ; Gelmukhanov *et al*., 1994 ; Meyer *et al*., 2014 ; Miyawaki *et al.*, 2017 ; Eckert *et al*., 2022). In this work, oxygen *K* edge RXES was undertaken for the MOF, MIL-53(Al), in order to selectively probe the carboxylate ligand orbitals participating in the metal-ligand interaction, both in the presence and absence of adsorbed water, and elucidate their role in modulating MOF vibrational dynamics responsible for pore breathing. RXES measurements were performed using the high-resolution soft x-ray emission spectrometer at the SPring-8 BL07LSU HORNET endstation (Harada *et al.*, 2012 ; Yamamoto *et al*., 2014). Electronic structure calculations on the benzenedicarboxylate anion ligand were undertaken to adequately account for the spectral features which exhibited change with temperature and water adsorption.

1. Results and Discussion
   1. X-ray absorption spectroscopy (XAS)


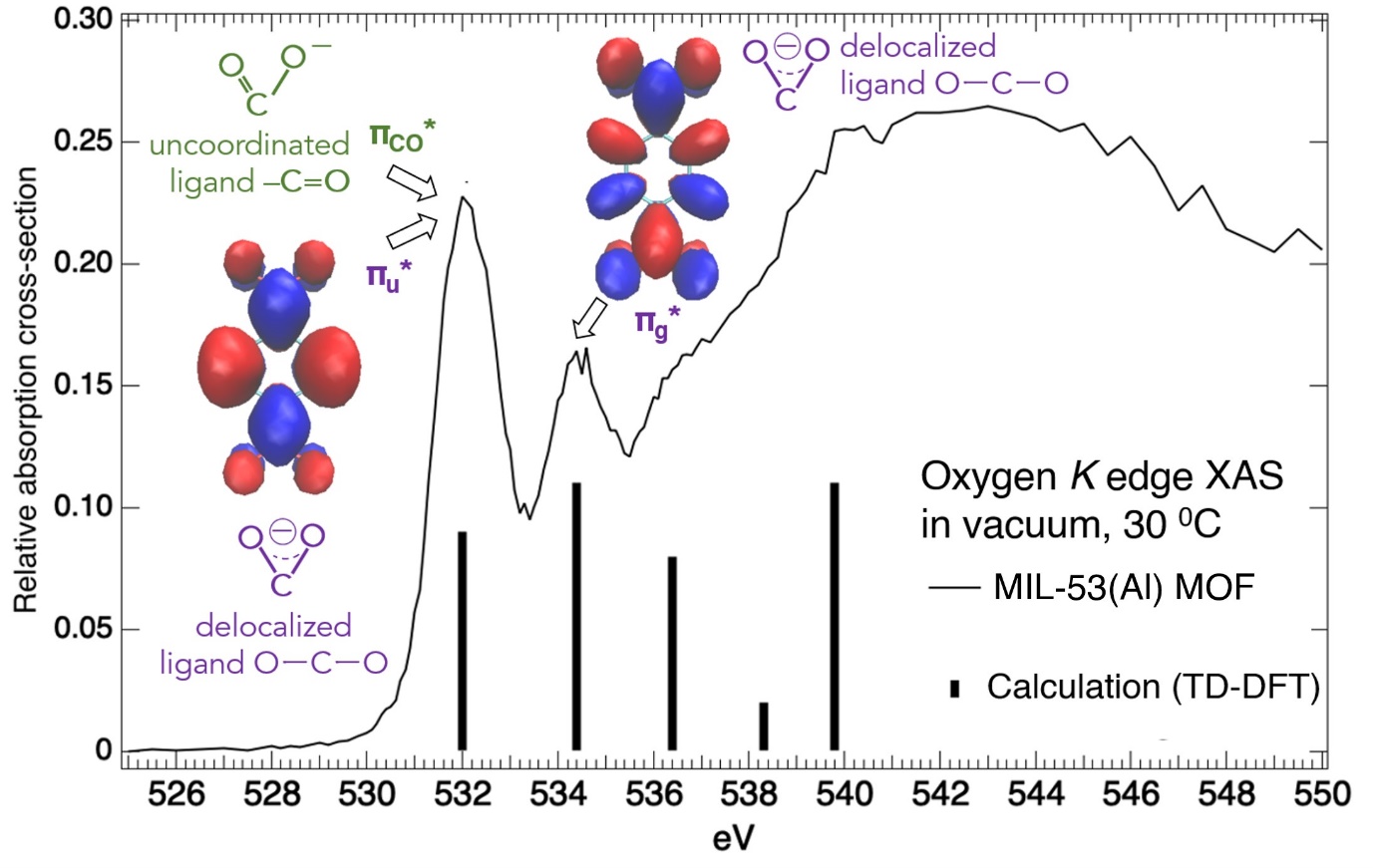


1. Oxygen *K* edge x-ray absorption (XAS) spectra for MIL-53(Al) MOF, in vacuum at 30 °C, compared with TD-DFT calculated XAS energies for the benzenedicarboxylate anion ligand, with the unoccupied orbitals π^*^*_u_,* π^*^*_g_,* and π^*^_CO_ assigned to the pre-edge features.

The oxygen *K* edge x-ray absorption spectra (XAS) of MIL-53(Al) MOF, in vacuum at 30 °C, showed two pre-edge features (Fig. 2) at 532.0 and 534.4 eV. Time-dependent density functional theory (TD-DFT) XAS calculations on the benzendicarboxylate (BDC) anion ligand suggested that the 532.0 and 534.4 eV pre-edge peaks arise from the unoccupied orbitals, π^*^*_u_*, and π^*^*_g_*, respectively. While both are derived from the same delocalized carboxylate COO antibonding π^*^ fragment orbital, they involve different benzene group orbitals which created the ~2.4 eV energy gap (Hennies *et al.*, 2007) between the π^*^*_u_*, and π^*^*_g_* states. Moreover, it is noted that the 532.0 eV pre-edge peak includes contribution from the antibonding π^*^_CO_ orbital for the localized carbonyl C=O group, as was observed in carboxylates and amino acids (Tokushima *et al*., 2009 ; Horikawa *et al*., 2009 ; Meyer *et al*., 2014 ; Eckert *et al*., 2022). Hence, RXES measurements were opted at 534.4 eV excitation, instead of at 532.0 eV, in order to probe the delocalized carboxylate COO units (Fig. 2) involved in bridging metal sites within the MOF structure. This minimizes the contribution of localized carbonyl C=O groups indicative of uncoordinated ligand sites in the subsequent RXES spectra.

- 1. Resonant x-ray emission spectroscopy


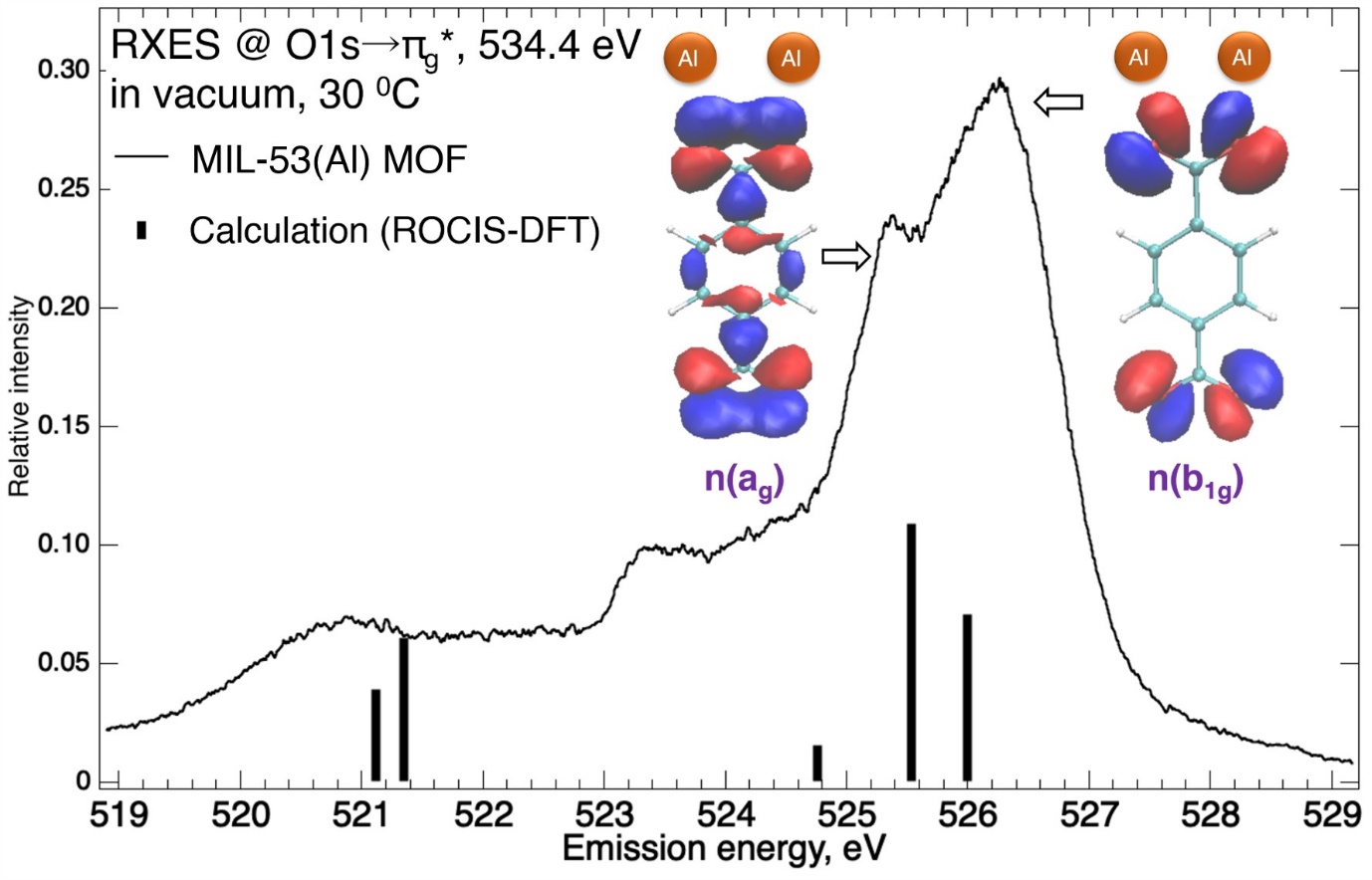


1. Resonant x-ray emission (RXES) spectra of MIL-53(Al) MOF at 534.4 eV excitation (O1*s* → π^*^*_g_*), in vacuum at 30 °C, compared with ROCIS-DFT calculated emission energies for the benzenedicarboxylate anion ligand, along with the occupied orbitals, *n*(*b*_1_*_g_*) and *n*(*a_g_*), derived from the ligand carboxylate oxygen lone pair orbitals in either antisymmetric *b*_1_*_g_* or symmetric *a_g_* configuration

Resonant x-ray emission (RXES) spectra (Fig. 3) at O1*s* → π^*^*_g_* excitation at 534.4 eV (Fig. 2) for the MIL-53(Al) MOF was measured in vacuum at 30 °C (Fig. 3). RXES calculations (Roemelt *et al.*, 2013), under restricted open configuration interaction with single excitations using DFT-derived orbitals (ROCIS-DFT) for the BDC anion ligand, suggested that the highest-lying emission features at 526.2 and 525.4 eV arise from the *n*(*b*_1_*_g_*) and *n*(*a_g_*) states (Fig. 3), respectively derived from the carboxylate COO oxygen in-plane lone pair orbitals. The calculated ~0.4 eV energy gap between these two states for the free BDC anion ligand was attributed to the difference in orbital overlap between the in-plane lone pair orbitals of the two oxygen atoms on the COO carboxylate group, in either an antisymmetric *b*_1_*_g_* or a symmetric *a_g_* configuration (Eckert *et al.*, 2022). In *n*(*b*_1_*_g_*), the antibonding-like interaction between the lone pair orbitals creates a region of reduced electron density between the carboxylate oxygen sites, along with a diffuse region of electron density distributed away from the oxygen sites and directed separately into the flanking metal centers (Fig. 3). In turn, in *n*(*a_g_*), the bonding-like interaction between the lone pair orbitals concentrates electron density within the region between the carboxylate oxygens, favoring shared interaction with the neighboring metal centers (Fig. 3). The deep-lying weak emission feature at ~521.4 eV was assigned to out-of-plane carboxylate π orbitals which delocalize into the neighboring benzene ring π system. The emission bands unaccounted for in the ligand RXES calculations are attributed to contributions from the oxide oxygens in the aluminum oxo centers (Ertan *et al*., 2017).

- 1. The role of temperature


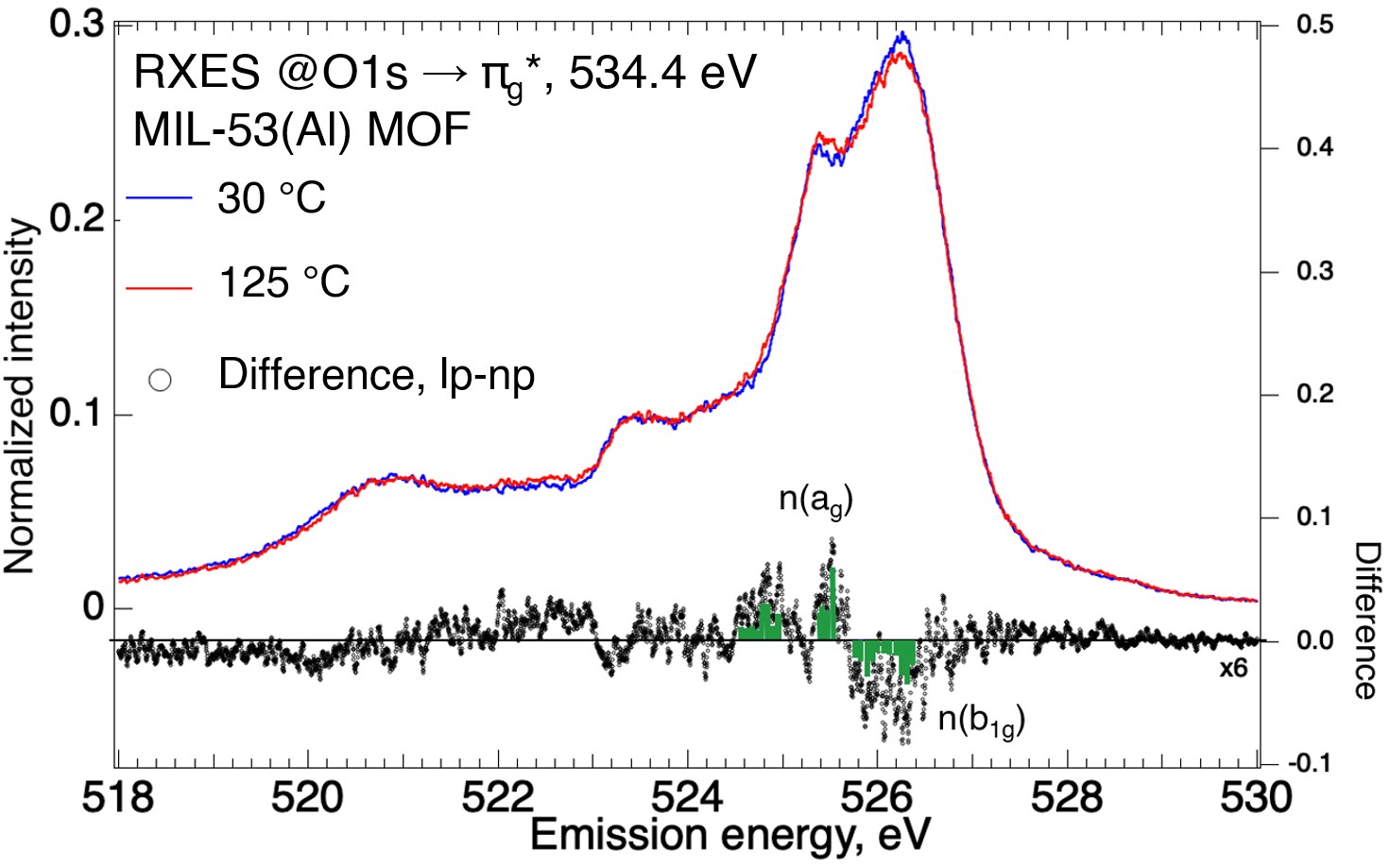


1. Resonant x-ray emission (RXES) spectra at 534.4 eV excitation (O1*s* → π^*^*_g_*) for the MIL-53(Al) MOF in vacuum, at 30 °C and 125 °C.

The temperature dependence of the RXES spectra at 534.4 eV excitation (Fig. 4) for the MIL-53(Al) MOF showed modulation of emission intensities for the highest-lying valence states, with a reduced emission at 526.2 eV (*n*(*b*_1_*_g_*)) compensated by an enhanced emission at 525.4 eV (*n*(*a_g_*)) upon temperature increase. Such electronic perturbation appears to be involved in the structural change accompanying the onset of pore breathing at higher temperatures (Loiseau *et al*., 2004 ; Liu *et al.*, 2008 ; Volkringer *et al*., 2009). This includes the slightly shorter carboxylate C-O bond inferred from the blue shift (SI, Fig. S1) for the stretching mode, and the modest increase in lattice constant (SI, Fig. S2) especially across the pore walls. The RXES spectra suggest that pore breathing, solely induced by temperature increase under vacuum, is accompanied by a modulation of orbital occupancy of *n*(*b*_1_*_g_*) and *n*(*a_g_*) states, as reflected in the change in their relative emission intensities.

1. Pseudo-Jahn-Teller mechanism for mode softening in a carboxylate MOF by tuning the occupancy of *n*(*b*_1_*_g_*) and *n*(*a_g_*) lone pair orbitals.

Ligand vibrational dynamics in carboxylate MOFs is closely related to the strength of metal-ligand (M−O) interaction (Andreeva *et al.*, 2020), with “loose” M−O bond populations preferred over “tight” ones, and stabilized by entropy at higher temperatures (Walker *et al*., 2010 : Wieme *et al*., 2018). Tuning the orbital population for the *n*(*b*_1_*_g_*) and *n*(*a_g_*) states (Fig. 5) is one mechanism towards modulating the strength of metal-ligand interaction, and the accompanying ligand vibrational dynamics and entropic stabilization. In this mechanism, *n*(*b*_1_*_g_*) orbital occupation at low temperature appears to be a precedent for carboxylate C−O bond inequivalence, as observed in the narrow-pore phase, as regions of electron density are directed towards the flanking metal centers separately due to the nodal plane between the carboxylate oxygens. In turn, *n*(*a_g_*) orbital occupation at high temperature appears to be a precedent for carboxylate C-O bond equivalence, as observed in the large-pore phase, owing to the shared interaction of the overlapping electron density regions with the neighboring metal centers.

A pseudo-Jahn-Teller (PJT) description of the modulation of orbital occupancy (Bersuker, 2013 ; Bersuker, 2021) is applied for the *n*(*b*_1_*_g_*) and *n*(*a_g_*) orbital populations of the MOF carboxylate upon temperature change. In the PJT mechanism, orbital populations can change via orbital mixing mediated by coupling these electronic states, Γ_el_(*b*_1_*_g_*) and Γ_el_(*a_g_*), to a vibrational mode, Γ_vib_(*b*_1_*_g_*), under the *D*_2_*_h_* point group symmetry of the BDC anion ligand, that satisfies the symmetry condition: Γ_el_(*b*_1_*_g_*) × Γ_el_(*a_g_*) × Γ_vib_(*b*_1_*_g_*) = Γ(*A_g_*). In particular, the carboxylate asymmetric stretching mode of the BDC ligand of *b*_1_*_g_* symmetry (Fig. 5) is taken to participate in this mechanism, as it is sensitive (SI, Fig. S1) to structural change in MOFs (Salazar *et al*., 2015 ; Hoffmann *et al*., 2018). Also, being an in-plane vibrational mode, the carboxylate *b*_1_*_g_* asymmetric stretching mode has a large spatial overlap with the in-plane *n*(*b*_1_*_g_*) and *n*(*a_g_*) lone pair orbitals being mixed, enhancing the PJT effect as a result (Sato *et al.*, 2006 ; Bersuker, 2013 ; Bersuker, 2021). Finally, it is remarked that while the microscopic mechanism of pore breathing in MIL-53 MOFs have been tackled on entropic and mechanical grounds (Walker *et al.*, 2010 ; Triguero *et al.*, 2011 ; Cockayne, 2017 ; Wieme *et al.*, 2018), the modulation of orbital occupancy (Fig. 5) elaborated in this work involves an earlier stage and a smaller scale of the structural phase transition, just at the onset of “loosening” or “tightening” the metal-ligand interaction that precedes the collective ligand motion needed for the drastic change in lattice structure during the breathing transition.

- 1. The role of water adsorption

The effect of water adsorption on the RXES spectra (Fig. 6) at 532.0 eV excitation (SI, Fig. S3) at 30 °C showed that upon MOF hydration, reduced emission at 525.4 eV is compensated by enhanced emission at 522.0 eV. These emission features at 525.4 and 522.0 eV derive from the carboxylate oxygen in-plane lone pair, and out-of-plane π orbitals, respectively (Horikawa *et al.*, 2009 ; Meyer *et al*., 2014 ; Eckert *et al.*, 2022). Such orbital modulation suggests how water adsorption can perturb the out-of-plane electron density by accessing deep-lying π orbitals, which was not observed (SI, Fig. S4) for pore breathing in vacuum solely induced by temperature. While a similar RXES behavior at 534.4 eV excitation was anticipated, this excitation energy already overlaps with the absorption pre-edge for the water molecule (Horikawa *et al.*, 2009 ; Meyer *et al*., 2014 ; Eckert *et al.*, 2022). Subtracting the contribution of adsorbed water would be difficult such that ultimately, RXES at 532.0 eV excitation was opted for in this case. It is remarked how such difference in orbital occupancy observed under vacuum and ambient conditions could provide alternative pathways for pore breathing, as exemplified in their distinct breathing kinetics, with a facile pore collapse under ambient conditions (Loiseau *et al*., 2004) compared to a severe hysteresis behavior under vacuum (Liu *et al.*, 2008).


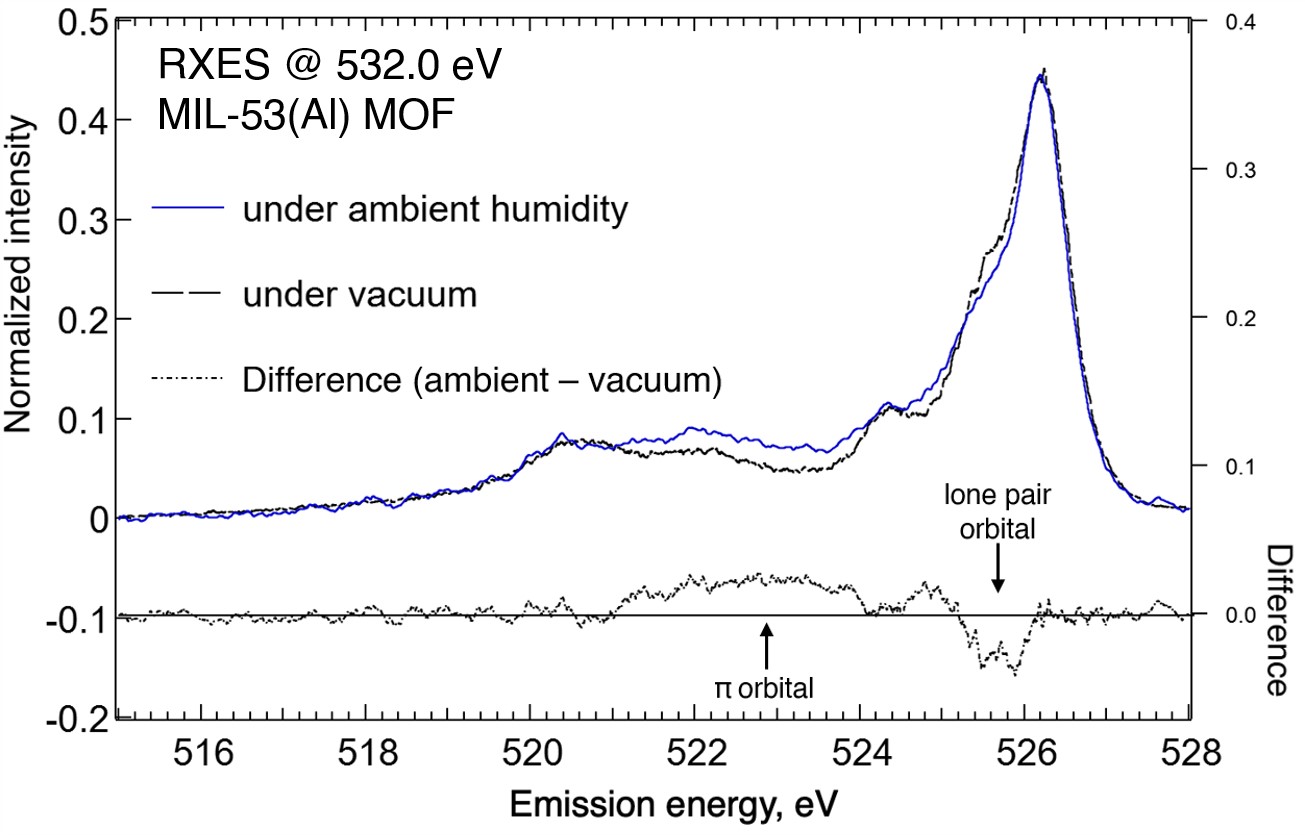


1. Resonant x-ray emission (RXES) spectra at 532.0 eV excitation for the MIL-53(Al) MOF at 30 °C under 60% relative humidity.
2. Conclusion

In summary, electronic perturbation at the ligand carboxylate accompanying pore breathing in the metal-organic framework MIL-53(Al) was observed by oxygen *K* edge resonant x-ray emission spectroscopy (RXES). Pore breathing in vacuum, solely induced by temperature, involved modulation of orbital occupancy of carboxylate oxygen in-plane lone pair orbitals in either an antisymmetric or a symmetric configuration. In turn, water adsorption into the MOF involved additional perturbation of out-of-plane π orbitals. More than a mere counterion, the carboxylate ligand bears an electronic structure motif that is intrinsically functional for driving structural change in MOFs. Tailoring the symmetry of the ligand carboxylate electronic states appears to be a potential route towards the design of novel functional MOFs with controllable structural transitions.

1. This work was supported by JSPS KAKENHI Grant Nos. JP19H05717 (Aquatic Functional Materials), JP22H05142 & JP22H05145 (Supraceramics), and JP19K20598. This work was in part carried out in Spring-8 BL07LSU (2018B7401, 2019A7401, 2019B7401) and in BL13XU (2023A1566, 2023A1774). We thank Prof. Kunihisa Sugimoto (Kinki University) for his assistance in powder XRD measurements.

References

Andreeva, A., Le, K., Chen, L., Kellman, M., Hendon, C., Brozek, C. (2020). *J. Am. Chem. Soc.* **142**,

19291-19299

Bara, D., Wilson, C., Mortel, M., Khusniyarov, M., Ling, S., Slater, B., Sproules, S., Forgan, R.

(2019). *J. Am. Chem. Soc.* **141**, 8346-8357

Bersuker, I. (2013). *Chem. Rev.* **113**, 1351-1390

Bersuker, I. (2021). *Chem. Rev.* **121**, 1463-1512

Cockayne, E. (2017). *J. Phys. Chem. C.* **121**, 4312-4317

Coudert, F., Jeffroy, M., Fuchs, A., Boutin, A., Mellot-Draznieks, C. (2008). *J. Am. Chem. Soc.* **130**,

14294-14302

Coudert, F., Boutin, A., Fuchs, A., Neimark, A. (2013). *J. Phys. Chem. Lett.* **4**, 3198-3205

Coudert, F., Ortiz, A., Haigis, V., Bousquet, D., Fuchs, A., Ballandras, A., Weber, G., Bezverkhyy, I.,

Geoffroy, N., Bellat, J., Ortiz, G., Chaplais, G., Patarin, J., Boutin, A. (2014). *J. Phys. Chem. C.* **118**, 5397- 5405

Eckert, S., Mascarenhas, E., Mitzner, R., Jay, R., Pietzsch, A., Fondell, M., da Cruz, V., Fohlisch, A.

(2022). *Inorg. Chem.* **61**, 10321-10328

Ertan, E., Kimberg, V., Gelmukhanov, F., Hennies, F., Rubensson, J., Schmitt, T., Strocov, V., Zhou,

K., Iannuzzi, M., Fohlisch, A., Odelius, M., Pietzsch, A. (2017). *Phys. Rev. B*. **95**, 144301

Gelmukhanov, F., Agren, H. (1994). *Phys. Rev. A.* **49**, 4378-4389

Grinnell, C., Samokhvalov. (2018) *Phys. Chem. Chem. Phys.* **20**, 26947-26956

Harada, Y., Kobayashi, M., Niwa, H., Senba, Y., Ohashi, H., Tokushima, T., Horikawa, Y., Shin, S.,

Oshima, M. (2012). *Rev. Sci. Instrum.* **83**, 01311

Hennies, F., Polyutov, S., Minkov, I., Pietzsch, A., Nagasono, M., Agren, H., Triguero, L.,

Piancastelli, M., Wurth, W., Gelmukhanov, F., Fohlisch, A. (2007). *Phys. Rev. A.* **76**, 032595

Hoffmann, A., Vanduyfhuys, L., Nevjestic, I., Wieme, J., Rogge, S., Depauw, H., Voort, P.,

Vrielinck, H., Speybroeck, V. (2018). *J. Phys. Chem. C.* **122**, 2734-2746

Horikawa, Y., Tokushima, T., Harada, Y., Takahashi , O., Chainani, A., Senba, Y., Ohashi, H.,

Hiraya, A., Shin, S. (2009). *Phys. Chem. Chem. Phys.* **11**, 8676-8679

Horike, S., Shimomura, S., Kitagawa, S. (2009). *Nat. Chem.* **1**, 695-704

Liu, Y., Her, J., Dailly, A., Ramirez-Cuesta, A., Neumann, D., Brown, C. (2008). *J. Am. Chem. Soc.*

**130**, 11813-11818

Loiseau, T., Serre, C., Huguenard, C., Fink, G., Taluelle, F., Henry, M., Bataille, T., Ferey, G. (2004).

*Chem. Eur. J.* **10**, 1373-1382

Meyer, F., Blum, M., Benkert, A., Hauschild, D., Nagarajan, S., Wilks, R., Andersson, J., Yang, W.,

Zharnikov, M., Bar, M., Heske, C., Reinert, F., Weinhardt, L. (2014). *J. Phys. Chem. B*. **118**, 13142-13150

Miyawaki, J., Suga, S., Fujiwara, H., Urasaki, M., Ikeno, H., Niwa, H., Kiuchi, H., Harada, Y. (2017).

*Phys. Rev. B*. **96**, 214420

Monson, P., McClain, W. (1970). *J. Chem. Phys*. **53**, 29-37

Roemelt, M., Maganas, D., DeBeer, S, Neese, F. (2013). *J. Chem. Phys.* **138**, 204101

Salazar, J., Weber, G., Simon, J., Bezverkhyy, I., Bellat J. (2015). *J. Chem. Phys.* **142**, 124702

Sato, T., Tokunaga, K., Tanaka, K. (2006). *J. Chem. Phys.* **124**, 024314

Shearer, G., Chavan, S., Ethiraj, J., Vitillo, J., Svelle, S., Olsbye, U., Lamberti, C., Bordiga, S., Lillerud, K. (2014). *Chem. Mater.* **26**, 4068-4071

Tokushima, T., Horikawa, Y., Harada, Y., Takahashi, O., Chainani, A., Senba, Y., Ohashi, H., Hiraya,

A., Shin, S. (2009). *Phys. Chem. Chem. Phys.* **11**, 1679-1682

Triguero, C., Coudert, F., Boutin, A., Fuchs, A., Neimark, A. (2011). *J. Phys. Chem. Lett.* **2**,

2033-2037

Volkringer, C., Loiseau, T., Guillou, N., Ferey, G., Elkaim, E., Vimont, A. (2009). *Dalton Trans.* **38**,

2241-2249

Walker, A., Civalleri, B., Slater, B., Mellot-Draznieks, F., Cora, C., Zicovich-Wilson, G., Roman-

Perez, G., Soler, J., Gale, J. (2010). *Angew. Chem. Int. Ed.* **49**, 7501-7503

Wieme, J., Lejaeghere, K., Kresse, G. Speybroeck, V. (2018). *Nat. Comm.* **9**, 4899

Yamamoto, S., Senba, Y., Tanaka, T., Ohashi, H., Hirono, T., Kimura, H., Fujisawa, M., Miyawaki,

J., Harasawa, A., Seike, S., Takahashi, S., Nariyama, N., Matsushita, T., Takeuchi, M., Ohata, T., Furukawa, Y., Takeshita, K., Goto, S., Harada, Y., Shin, S., Kitamura, H.,, Kakizaki, A., Oshima, M., Matsuda, I. (2014). *J. Synchrotron Rad.* **21**, 352-365

Supporting Information


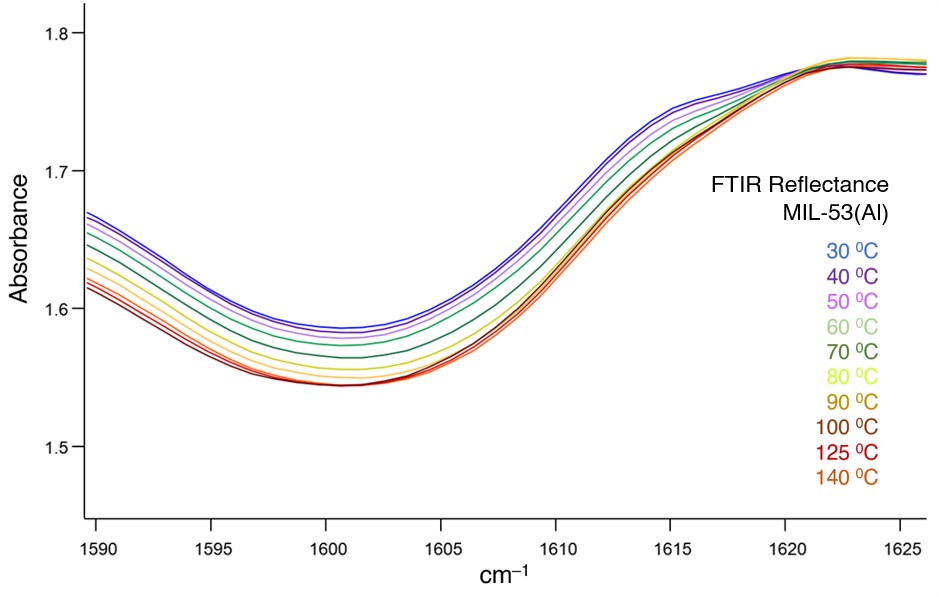


1. Variable temperature FTIR spectra of MIL-53(Al) MOF pellet sample measured by reflectance mode. A blue shift is noted from 1614 cm^-1^ to 1622 cm^-1^, by ~8 cm^-1^ as temperature was increased.


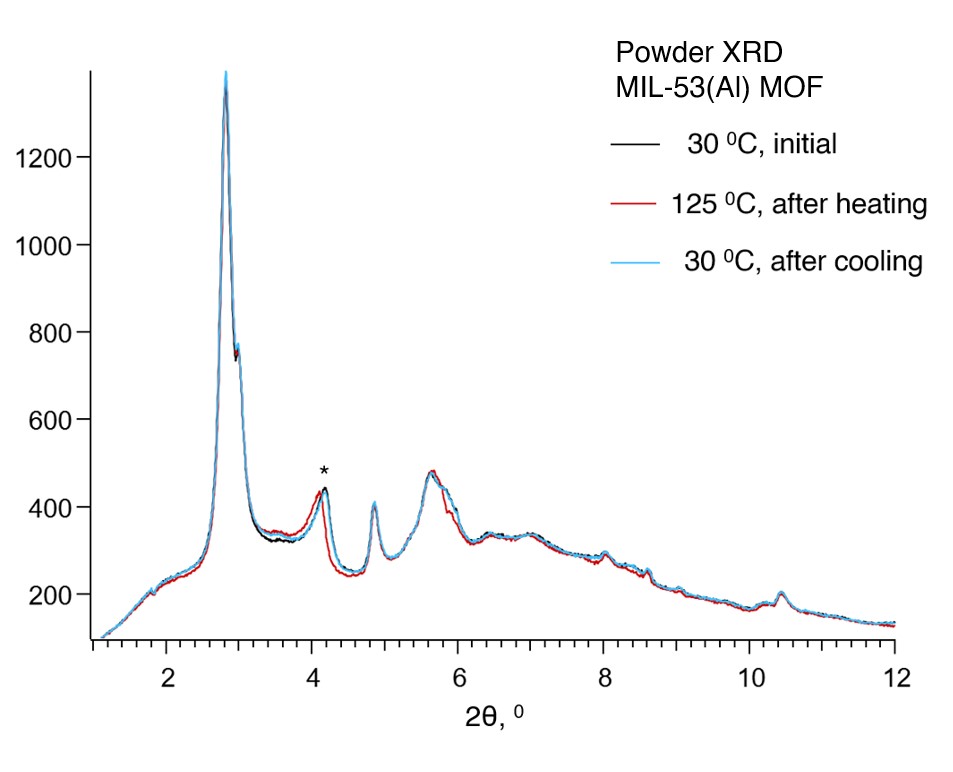


1. Variable temperature powder XRD profiles for MIL-53(Al) MOF under vacuum (~ 0.5 Pa). A shift in the diffraction peak (*) at 2*θ* = 4.2° to 4.1°, upon heating, was taken to indicate the onset of pore expansion due to increase in the lattice constant along the 110 plane (Volkringer *et al*., 2009), which occurs parallel to the rhombohedral faces of the narrow-pore form. Recovery of the XRD profile upon cooling back to the initial temperature was taken to indicate that the effect of desorption of pre-existing guest species within the nanopores of the MOF sample is minimal. Crystal structures were visualized using VESTA (Monma *et al*., 2008).


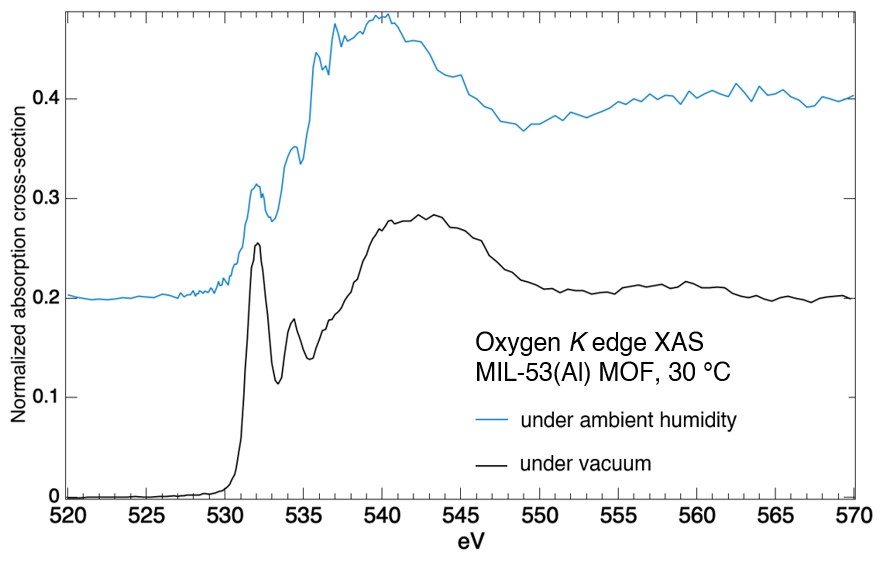


1. Oxygen *K* edge XAS spectra for MIL-53(Al) MOF at 30 °C under ambient (60% relative humidity) and vacuum conditions.


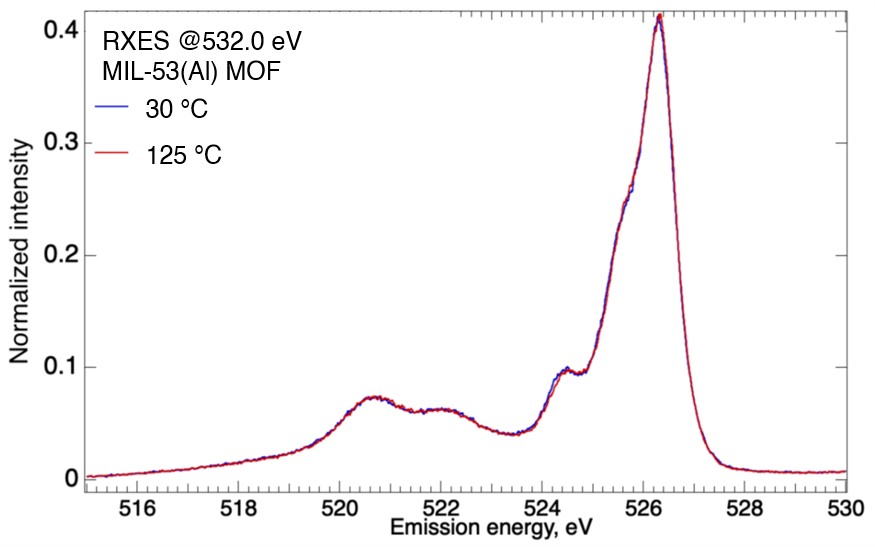


1. Resonant x-ray emission (RXES) spectra at 532.0 eV excitation for MIL-53(Al) MOF at 30 °C and 125 °C in vacuum.

1. Experimental setup for ambient RXES and XAS measurements at SPring-8 BL07LSU HORNET endstation.
2. Experimental detail
   1. Sample preparation and characterization

The metal-organic framework MIL-53(Al) (BASF Basolite A100, Sigma-Aldrich Co. LLC, Japan) was used for measurements as received. For vacuum measurements, the MOF samples were lightly pressed into a pellet. For ambient measurements, the MOF sample was drop-casted onto a a SiC(150 nm)/Cr(3 nm)/Au(11 nm) membrane (NTT Advanced Technology Co. Japan), with a 0.3 × 3 mm^2^ window transparent to soft x-rays (Fig. S5), as routinely employed in SPring-8 BL07LSU for ambient measurements. First, the MOF powder was suspended in distilled water. Then, a few drops of the suspension were laid onto the SiC membrane (Drisdell *et al*., 2013), which was then heated at 60 ^0^C to dry out the sample. Under these conditions, hydrolysis or phase transformation should be negligible (Kang *et al*., 2011).

Variable temperature Fourier transform infrared spectroscopy (FTIR) measurements in reflectance mode were performed on a pellet sample using a JASCO FTIR 6600 spectrometer (JASCO, Japan). Variable temperature powder x-ray diffraction (XRD) measurements under vacuum (< 0.5 Pa) were performed in SPring-8 BL13XU, at an x-ray wavelength of 0.495656 Å. Powder samples were loaded into 0.5 mm glass capillary tubes which were constantly rotated during measurement. Sample heating was achieved by subjecting the sample-loaded capillary tubes to a flow of warm air which has been previously calibrated for sample temperature.

- 1. XAS and RXES measurements

Oxygen *K* edge x-ray absorption (XAS) and resonant x-ray emission (RXES) measurements were performed using the high-resolution soft x-ray emission spectrometer HORNET at SPring-8 BL07LSU. For XAS measurements, an incident energy resolution of 0.1 eV was observed. To minimize radiation damage, photon flux was limited by adjusting an exit slit opening and moving the sample position away from the beam focus. Partial fluorescence yield (PFY) XAS spectra were acquired using a XR-100SDD silicon drift detector (Amptek, USA). In turn, RXES measurements were done at 90^0^ scattering geometry, with a < 0.15 eV emission energy resolution. The pellet sample under vacuum was continuously moved at ~1.6 μm s^-1^ during spectral acquisition in order to minimize radiation damage, for a ~ 5 × 30 μm^2^ beam size. In turn, for the dropcast sample in the ambient measurements, a position in the membrane window was irradiated by 5 s at most, before moving the window by ~10 μm.

For vacuum measurements, temperature control was achieved using a cryostat system. Temperature was confirmed at the pellet sample surface directly hit by the x-rays using an alumel-chromel K-type thermocouple. Ambient measurements were performed using a liquid flow cell setup (Fig. S5) routinely employed at SPring-8 BL07LSU HORNET. Sample environment was maintained at around 60% relative humidity and 30 °C to simulate the conditions in which the hydrated MOF was previously reported (Loiseau *et al*., 2004). Humidity was controlled with a steam generator (HUM-1, Rigaku Co., Japan), and monitored with a TRH-7X humidity-temperature sensor (Shinyei Co. Japan).

1. Computational detail

Single-point electronic structure calculations were performed for the MOF ligand benzenedicarboxylate (BDC) anion, C_6_H_4_(COO)_2_^2-^, under a *D*_2h_ point group symmetry using the ORCA quantum chemistry package (Neese, 2012). XAS calculations were done under time-dependent density functional theory (TD-DFT), using the B3LYP functional (Becke, 1993 ; Stephens *et al*., 1994), and the def2-TZVP Ahlrichs basis set (Weigend *et al*., 2005). RXES calculations were performed using restricted open-shell configuration interaction with single excitations using DFT-derived orbitals (ROCIS-DFT) from the B3LYP functional (Becke, 1993 ; Stephens *et al*., 1994) and def2-TZVP Ahlrichs basis set (Weigend *et al*., 2005), and adopting previously optimized CI matrix terms for incorporating the DFT-derived orbitals into the ROCIS calculation. The def2-TZVP/C auxiliary basis set (Chmela *et al*., 2018) was used for the RI approximation of Coulomb and exchange integrals. Calculated XAS and RXES energies were shifted by +15 eV and +14 eV, respectively to align with the experimental energies.

References

Becke, A. (1993). *J. Chem. Phys.* **98**, 1372-1377

Chmela, J., Harding, M. (2018). *Mol. Phys.* **116**, 1523-1538

Drisdell, W., Poloni, R., McDonald, T., Long, J., Smit, B., Neaton, J., Prendergast, D., Kortright, J.

(2013). *J. Am. Chem. Soc.* **135**, 18183-18190

Kang, I., Khan, N., Haque, E., Jhung, S. (2011). *Chem. Eur. J.* **17**, 6437-6442

Loiseau, T., Serre, C., Huguenard, C., Fink, G., Taluelle, F., Henry, M., Bataille, T., Ferey, G. (2004).

*Chem. Eur. J.* **10**, 1373-1382

Monma, K., Izumi, F. (2008). *J. Appl. Cryst*. **41**, 653-658

Neese, F. (2012). *WIREs Computational Mol. Sci.* **2**, 73-78

Stephens, J., Devlin, F., Chablowski, C., Frisch, M. (1994). *J. Phys. Chem.* **98**, 11623-11627

Volkringer, C., Loiseau, T., Guillou, N., Ferey, G., Elkaim, E., Vimont, A. (2009). *Dalton Trans.* **38**,

2241-2249

Weigend, F., Ahlrichs, R. (2005). *Phys. Chem. Chem. Phys*. **7**, 3297-3305
